# Rationale and evidence for the use of new beta-lactam/beta-lactamase inhibitor combinations and cefiderocol in critically ill patients

**DOI:** 10.1186/s13613-023-01153-6

**Published:** 2023-07-18

**Authors:** François Barbier, Sami Hraiech, Solen Kernéis, Nathanaël Veluppillai, Olivier Pajot, Julien Poissy, Damien Roux, Jean-Ralph Zahar, On behalf of the French Intensive Care Society

**Affiliations:** 1 Médecine Intensive Réanimation, Centre Hospitalier Régional d’Orléans, 14, Avenue de l’Hôpital, 45000 Orléans, France; 2 Institut Maurice Rapin, Hôpital Henri Mondor, Créteil, France; 3 Médecine Intensive Réanimation, Hôpital Nord, Assistance Publique – Hôpitaux de Marseille, and Centre d’Études et de Recherche sur les Services de Santé et la Qualité de Vie, Université Aix-Marseille, Marseille, France; 4 Équipe de Prévention du Risque Infectieux, Hôpital Bichat-Claude Bernard, Assistance Publique – Hôpitaux de Paris, and INSERM/IAME, Université Paris Cité, Paris, France; 5 Réanimation Polyvalente, Hôpital Victor Dupouy, Argenteuil, France; 6 Médecine Intensive Réanimation, Centre Hospitalier Universitaire de Lille, Inserm U1285, Université de Lille, and CNRS/UMR 8576 - UGSF - Unité de Glycobiologie Structurale et Fonctionnelle, Lille, France; 7 DMU ESPRIT, Médecine Intensive Réanimation, Hôpital Louis Mourier, Assistance Publique – Hôpitaux de Paris, Colombes, and INSERM/CNRS, Institut Necker Enfants Malades, Université Paris Cité, Paris, France; 8 Département de Microbiologie Clinique, Hôpital Avicenne, Assistance Publique – Hôpitaux de Paris, Bobigny and INSERM/IAME, Université de Paris, Paris, France

**Keywords:** Cefiderocol, Ceftolozane–tazobactam, Ceftazidime–avibactam, Meropenem–vaborbactam, Imipenem–relebactam, Aztreonam, Enterobacterales, *Pseudomonas aeruginosa*, Carbapenem resistance, Intensive care unit

## Abstract

**Background:**

Healthcare-associated infections involving Gram-negative bacteria (GNB) with difficult-to-treat resistance (DTR) phenotype are associated with impaired patient-centered outcomes and poses daily therapeutic challenges in most of intensive care units worldwide. Over the recent years, four innovative β-lactam/β-lactamase inhibitor (BL/BLI) combinations (ceftolozane–tazobactam, ceftazidime–avibactam, imipenem–relebactam and meropenem–vaborbactam) and a new siderophore cephalosporin (cefiderocol) have been approved for the treatment of certain DTR-GNB infections. The literature addressing their microbiological spectrum, pharmacokinetics, clinical efficacy and safety was exhaustively audited by our group to support the recent guidelines of the French Intensive Care Society on their utilization in critically ill patients. This narrative review summarizes the available evidence and unanswered questions on these issues.

**Methods:**

A systematic search for English-language publications in PUBMED and the Cochrane Library database from inception to November 15, 2022.

**Results:**

These drugs have demonstrated relevant clinical success rates and a reduced renal risk in most of severe infections for whom polymyxin- and/or aminoglycoside-based regimen were historically used as last-resort strategies—namely, ceftazidime–avibactam for infections due to *Klebsiella pneumoniae* carbapenemase (KPC)- or OXA-48-like-producing Enterobacterales, meropenem–vaborbactam for KPC-producing Enterobacterales, ceftazidime–avibactam/aztreonam combination or cefiderocol for metallo-β-lactamase (MBL)-producing Enterobacterales, and ceftolozane–tazobactam, ceftazidime–avibactam and imipenem–relebactam for non-MBL-producing DTR *Pseudomonas aeruginosa*. However, limited clinical evidence exists in critically ill patients. Extended-infusion scheme (except for imipenem–relebactam) may be indicated for DTR-GNB with high minimal inhibitory concentrations and/or in case of augmented renal clearance. The potential benefit of combining these agents with other antimicrobials remains under-investigated, notably for the most severe presentations. Other important knowledge gaps include pharmacokinetic information in particular situations (e.g., pneumonia, other deep-seated infections, and renal replacement therapy), the hazard of treatment-emergent resistance and possible preventive measures, the safety of high-dose regimen, the potential usefulness of rapid molecular diagnostic tools to rationalize their empirical utilization, and optimal treatment durations. Comparative clinical, ecological, and medico-economic data are needed for infections in whom two or more of these agents exhibit in vitro activity against the causative pathogen.

**Conclusions:**

New BL/BLI combinations and cefiderocol represent long-awaited options for improving the management of DTR-GNB infections. Several research axes must be explored to better define the positioning and appropriate administration scheme of these drugs in critically ill patients.

**Supplementary Information:**

The online version contains supplementary material available at 10.1186/s13613-023-01153-6.

## Introduction

Carbapenems stand as the main option for the treatment of severe infections due to Gram-negative bacteria (GNB) exhibiting resistance to broad-spectrum penicillins and cephalosporins [1–3]. Yet, the dissemination of carbapenem-resistant GNB, either at an endemic state or during outbreak phenomenon, now poses daily therapeutic challenges in most of intensive care units (ICU) worldwide [4–8]. Infections involving carbapenem-resistant GNB are associated with a substantial rise in fatality rates, length of hospital stay and costs of care when compared to those caused by carbapenem-susceptible isolates [9–14]. These impaired outcomes may result from a higher likelihood of inadequate empirical therapy, toxicity attributable to historical last-resort agents (e.g., polymyxin-related acute kidney injury), and the frailty of individuals in whom such conditions predominantly occur [15]. In addition, most of carbapenem-resistant GNB are resistant to other antimicrobial classes, further complicating the management of infected patients—hence, co-resistance to carbapenems, all other first-line β-lactams and fluoroquinolones is consensually defined as difficult-to-treat resistance (DTR) [1–3].

Over the recent years, four innovative β-lactam/β-lactamase inhibitor (BL/BLI) combinations (namely, ceftolozane–tazobactam, ceftazidime–avibactam, imipenem–relebactam and meropenem–vaborbactam) and a new siderophore cephalosporin (cefiderocol) have been introduced and approved for the treatment of certain DTR-GNB infections. The literature addressing their microbiological spectrum, pharmacokinetics, clinical efficacy and safety in critically ill patients has been exhaustively audited by our group to support the guidelines of the French Intensive Care Society, elaborated during a consensus conference held in Paris on November 30, 2022 and published in this issue of *Annals of Intensive Care*. In this narrative review, we summarize the available evidence and knowledge gaps on these questions, with a focus on DTR-GNB infections.

## Methods

We systematically searched PubMed and the Cochrane Library database from inception to November 15, 2022. The search terms are listed in Additional file 1 of this article. We manually searched the reference lists of the included studies and systematic reviews to select additional relevant articles. Studies published in languages other than English were not retained.

## Current epidemiology of DTR-GNB in critically ill patients

Critically ill patients present a marked predisposition for DTR-GNB infection as a combined result of massive exposure to broad-spectrum antimicrobials damaging the resident microbiotas and their inherent colonization resistance functions, and repeated opportunities for cross-transmission ensuing from invasive procedures and prolonged hospitalization [16, 17]. Enterobacterales (primarily *Klebsiella pneumoniae* and *Escherichia coli*), *Pseudomonas aeruginosa*, *Acinetobacter baumannii* and *Stenotrophomonas maltophilia* account for virtually all healthcare-associated DTR-GNB infections in the ICU.

Carbapenem resistance in Enterobacterales depends almost exclusively on the acquisition and expression of plasmid-borne carbapenemases belonging to the A (mostly *Klebsiella pneumoniae* carbapenemase [KPC]), B (metallo-β-lactamases [MBL], especially New-Delhi MBL [NDM]) or D (oxacillinases, mainly OXA-48-like carbapenemases) classes of the Ambler’s scheme (Table 1) [18, 19]. The prevalence of carbapenemase-producing Enterobacterales (CPE) is increasing globally, with a marked trend for MBL-producing isolates—this pandemic shows large geographical disparities, with a North/South gradient and higher prevalence in low- and middle-income countries (Table 2) [8, 20–23]. Endemic states have been reached in Italy, Greece and the United States for KPC producers, in the India/Pakistan region for NDM producers, and in the India/Pakistan and Mediterranean regions for OXA-48 producers; however, sporadic outbreaks are now regularly reported in other areas, including Latin America, Oceania, Africa and Northern Europe [24]. In 2017, in the 37 European countries contributing to the eCDC surveillance program, the prevalence of carbapenem resistance among invasive isolates of *K. pneumoniae* and *E. coli* ranged from 0 to 65% and from 0% to 1.6%, respectively; 16 (43%) countries reported regional or interregional spread of CPE while four countries (Greece, Italy, Turkey and Malta) declared an endemic situation [25]. In France, CPE infections remain infrequent, with less than 1000 cases reported annually [26], in line with a low prevalence of colonization—indeed, in a recent study including 2396 hospitalized patients, the rate of intestinal carriage of CPE was still 1.2% [27].

**Table 1:** In vitro activity of novel β-lactam/β-lactamase inhibitor combinations and cefiderocol against carbapenem-resistant Gram-negative bacteria

Main mechanisms of carbapenem resistance	Enterobacterales			*Pseudomonas aeruginosa*	*Acinetobacter baumannii*	*Stenotrophomonas maltophilia*
	Class A carbapenemase (KPC)	Class D carbapenemase (OXA-48-like^a^)	Class B carbapenemase (MBL^b^)	*OprD2* mutation Efflux^c^ MBL^d^	OXA^e^	Chromosomal MBL
Ceftolozane–tazobactam	–	–	–	+++ 75%-90% ^f^	–^g^	–^g^
Ceftazidime–avibactam	+++ 96%-99%	+++ 96%-99%	–	++ 60%-70%	–^g^	–^g^
Ceftazidime–avibactam plus aztreonam	+++ 96–99%	+++ 96%-99%	+++ > 90%	± (MBL) 0–25%	–^g^	++^h^ ~ 85%
Meropenem–vaborbactam	+++ 95–99%	–	–	–	–	–^g^
Imipenem–relebactam	+++ 88%-95%	±	–	++ 70%-90%	–	–^g^
Cefiderocol	+++ 84–91%	+++ 88–93%	++ VIM: 79%-81% NDM: 41%-51%	+++ > 90%	+++^i^ MIC ≤ 2 mg/L for > 90% of isolates	+++^i^ MIC ≤ 2 mg/L for > 90% of isolates

From references [31–35, 38, 40, 42–45, 50, 51, 54, 56–60, 63, 64, 66, 68, 69, 74, 75]

Susceptibility profiles are indicated for carbapenem-resistant isolates only

KPC: *Klebsiella pneumoniae* carbapenemase; MBL: metallo-β-lactamase; NDM: New-Delhi MBL; MIC: minimal inhibitory concentration

^a^OXA-48 and derivatives (e.g., OXA-181 and OXA-232)—note that these β-lactamases hydrolyze penicillins and carbapenems but not broad-spectrum cephalosporins; ^b^NDM is the main MBL type in Enterobacterales (other types such as VIM are less common); ^c^main efflux pump systems implicated in carbapenem resistance in *P. aeruginosa* are MexAB-OprM and MexEF-OprN; ^d^various MBL types (VIM, IMP, NDM, SPM)—other carbapenemase types are occasionally documented in *P. aeruginosa*, including class A (KPC and GES) and C (OXA-48-like) carbapenemases; ^e^mainly OXA-23-, OXA-24/40-, and OXA-58–like enzymes; ^f^ceftolozane–tazobactam is not active against carbapenemase-producing isolates of *P. aeruginosa*; ^g^intrinsic resistance or limited susceptibility (species); ^h^activity resting on the combination of aztreonam and avibactam (see the text for explanations); ^i^ no defined EUCAST breakpoint (insufficient data)

**Table 2:** Prevalence of carbapenem resistance among Gram-negative bacteria isolated from clinical samples in selected national and international surveillance networks

Surveillance network	CHINET	CDC/NHSN	SENTRY	SENTRY	SENTRY	EB-2	EARS-Net	SPIADI	SPIADI
Geographical area	China	USA	USA	Western Europe	Eastern Europe	Worldwide	Europe	France	France
Sample collection, period	2016	2015–2017	2016–2019	2016–2019	2016–2019	2019–2020	2021	2021	2021
Infection type	HA-BSI	DA-HAI	Pneumonia ^a^	Pneumonia^a^	Pneumonia^a^	HA-BSI (ICU)	All^b^	VAP (ICU)	HA-CR-BSI
References	[20]	[21]	[22]	[22]	[22]	[23]	[8]	[211]	[211]
Enterobacterales *Escherichia coli* *Klebsiella pneumoniae*	– 1.6% 42.9%	– 0.7% 6.9%	– 0.5% 4.7%	– 0.4% 8.7%	– 0.5% 17.5%	– 7.4% 37.8%	– 0.2% 11.7%	– – –	0.6% – –
*Pseudomonas aeruginosa*	24.3%	20.7%	25.2%	23.1%	51.7%	33.2%	18.1%	20.8%	9.0%
*Acinetobacter baumannii*	57.7%	43.2%	41.2%	54.2%	89.6%	84.6%	39.9%	22.8%	–

HA: hospital-acquired; BSI: bloodstream infection; DA-HAI: device-associated healthcare-associated infection; ICU: intensive care unit; VAP: ventilator-associated pneumoniae; CR: catheter-related

^a^Community-onset pneumonia requiring hospitalization and hospital-acquired pneumonia (pooled); ^b^community-onset infections and hospital-acquired infections (pooled)

In *Pseudomonas aeruginosa*, carbapenem resistance rests on both plasmid-borne carbapenemases (mostly MBL such as VIM, IMP, NDM or SPM) and chromosomal mutations leading to over-expression of efflux pump systems or impermeability through porin D2 alterations [18]. The overall prevalence of carbapenem resistance in invasive isolates of *P. aeruginosa* fluctuates between 10 and 20% in most geographic areas [8, 20, 22, 23], with a variable proportion of DTR phenotypes that may locally increase owing to outbreaks related to certain high-risk clones (Table 2) [28, 29]. Next, as for Enterobacterales, carbapenem resistance in *Acinetobacter baumannii* is predominantly driven by plasmid-borne carbapenemases, with OXA-23 as the leading type [19]. Rates of carbapenem resistance in invasive isolates of *A. baumannii* have reached critical levels in the Mediterranean area, South-East Asia and, to a lesser extent, Southern Europe (Table 2). In a recent global study including 350 critically ill patients with hospital-acquired bloodstream infection due to *A. baumannii*, 296 (84.6%) were infected by CR isolates [23]. Lastly, *Stenotrophomonas maltophilia* expresses a chromosomally encoded MBL that confers intrinsic carbapenem resistance to the species [18]. Infections due to this pathogen mostly occur in critically ill and/or immunocompromised hosts, especially in those with prior exposure to carbapenems or other broad-spectrum β-lactams [30]. Resistance to both cotrimoxazole and fluoroquinolones may pragmatically correspond to a DTR phenotype though there is no consensual definition for this species.

## Bacterial spectrum of cefiderocol and new BL/BLI combinations

### In vitro activity against carbapenemase-producing Enterobacterales

Avibactam is a potent inhibitor of serine-β-lactamases (that is, Ambler’s classes A, C and D); therefore, ceftazidime–avibactam is active in vitro against 96–99% of KPC- and OXA-48-like-producing Enterobacterales (Table 1) [31–35]. Yet, resistance to ceftazidime–avibactam may emerge following mutations in KPC-encoding genes (mainly *bla*_KPC-2_ and *bla*_KPC-3_) or genes encoding outer membrane proteins (OmpK35-37), especially when associated with a high number of *bla*_KPC-2_ copies [36–39]. MBL-producing Enterobacterales are resistant to ceftazidime–avibactam since avibactam has no inhibitory effect on these enzymes. However, the combination of ceftazidime–avibactam plus aztreonam is active against around 80% of MBL-producing Enterobacterales [40], aztreonam being not hydrolyzed by MBL and avibactam inhibiting the other β-lactamases commonly co-produced by such isolates (e.g., hyperproduced AmpC cephalosporinases, extended-spectrum β-lactamases [ESBL], or class A carbapenemases such as KPC, all including aztreonam in their hydrolysis spectrum) [41, 42].

Relebactam and vaborbactam inhibit class A β-lactamases, without effect on MBL or OXA carbapenemases [43]. Imipenem–relebactam and meropenem–vaborbactam are active in vitro against 88–95% and 95–99% of KPC-producing Enterobacterales, respectively [43–45]. Certain KPC variants and ESBL co-expression have been linked with a rise in minimal inhibitory concentrations (MIC) of imipenem–relebactam while over-expression of *bla*_KPC_ may increase those of meropenem–vaborbactam; in addition, porin mutations (OmpK35, OmpK 36) can reduce the activity of both drugs [46–48]. Importantly, these combinations remain occasionally active against isolates producing KPC-2 or KPC-3 variants with reduced susceptibility to the inhibitory effect of avibactam [45, 49].

Cefiderocol is a poor substrate for all β-lactamase classes and shows in vitro activity against 84–81%, 88–93%, 79–81% and 41–51% of KPC-, OXA-48-, VIM- and NDM-producing Enterobacterales, ESBL co-expression and porin mutations being associated with a reduced activity of the drug [50, 51]. Also, the inoculum effect—that is, an elevation of MIC values for high bacterial concentrations [52]—appears more pronounced with cefiderocol than with other new agents though the clinical significance of this finding is not yet elucidated [53].

The MIC cut-off values of novel BL/BLI combinations and cefiderocol for Enterobacterales, as defined by the European Committee on Antimicrobial Susceptibility Testing (EUCAST), are exposed in Table 3. Importantly, ceftolozane is hydrolyzed by all described carbapenemases, none of which being inhibited by tazobactam; therefore, ceftolozane–tazobactam exerts no activity against CPE [54, 55].

**Table 3:** EUCAST MIC cut-off values defining susceptibility to new β-lactam/β-lactamase inhibitor combinations and cefiderocol for Enterobacterales, *Pseudomonas aeruginosa*, *Acinetobacter baumannii* and *Stenotrophomonas maltophilia*

	Ceftolozane–tazobactam	Ceftazidime–avibactam	Imipenem–relebactam	Meropenem–vaborbactam	Cefiderocol
Enterobacterales	≤ 2 mg/L	≤ 8 mg/L	≤ 2 mg/L	≤ 8 mg/L	≤ 2 mg/L
*Pseudomonas aeruginosa*	≤ 4 mg/L	≤ 8 mg/L	≤ 2 mg/L	≤ 8 mg/L	≤ 2 mg/L
*Acinetobacter baumannii*	IR	NA	≤ 2 mg/L	≤ 2 mg/L ^a^	NA
*Stenotrophomonas maltophilia*	IR	NA	IR	IR	NA

Source: www.eucast.org/clinical_breakpoints

EUCAST: European Committee on Antimicrobial Susceptibility Testing; MIC: minimal inhibitory concentration; IR: intrinsic resistance; NA: non-appropriate (species with intrinsically weak susceptibility to the considered drug or insufficient data to define a MIC cut-off value)

^a^MIC cut-off for meropenem

### In vitro activity against carbapenem-resistant non-fermenting GNB

Ceftolozane circumvents two major mechanisms of β-lactam resistance in *P. aeruginosa*—namely, efflux and AmpC-mediated hydrolysis [56]. This fifth-generation cephalosporin, independently of its association with tazobactam, is active in vitro against 75–90% of non-carbapenemase-producing carbapenem-resistant *P. aeruginosa* isolates and, more globally, against 40–80% of DTR isolates within this species [54, 57–61]. Resistance to ceftolozane–tazobactam in *P. aeruginosa* depends on either certain plasmid-borne β-lactamases (e.g., MBL, OXA-14, OXA-19, OXA-35, GES-9, or PER-1) or extreme over-expression of chromosomal AmpC [62]. *A. baumannii* and *S. maltophilia* are intrinsically resistant to ceftolozane.

Both avibactam and relebactam inhibit chromosomal AmpC in *P. aeruginosa*. Ceftazidime–avibactam and imipenem–relebactam are active against 65%-85% of DTR isolates of *P. aeruginosa* [57–59, 63, 64]. Avibactam does not restore ceftazidime activity in MBL-producing isolates or in those with over-expressed efflux pump systems for which ceftazidime is a substrate. Relebactam may restore imipenem activity is isolates with mutated D2 porin and derepressed AmpC, likely due to the limited but significant hydrolysis of imipenem by this enzyme [65]. These two combinations lack activity against carbapenemase-producing isolates of *A. baumannii* and against *S. maltophilia*, this later species being intrinsically poorly susceptible to ceftazidime and resistant to imipenem [18]. Meropenem–vaborbactam is not active against meropenem-resistant isolates of *P. aeruginosa* or *A. baumannii* due to the lack of inhibitory effect of vaborbactam on mechanisms of meropenem resistance in these species (that is efflux, impermeability, or carbapenemase production).

Cefiderocol is active in vitro against 90% to 95% of carbapenem-resistant isolates of *P. aeruginosa* [66–69]. No MIC threshold of cefiderocol is currently defined by the EUCAST for *A. baumannii* and *S. maltophilia*; however, more than 90% of isolates within these species show MIC values below 2 mg/L (i.e., the cut-off value for Enterobacterales and *P. aeruginosa*) [69].

A key-point is that the multiplicity of potentially involved resistance mechanisms makes unpredictable the activity of new β-lactams in DTR *P. aeruginosa*. Indeed, a substantial proportion of isolates exhibiting resistance to one agent remains susceptible to others, which implies to perform susceptibility tests for all novel BL/BLI combinations and cefiderocol in isolates with such phenotypes [70]. Of note, gradient test strips are not accurate to measure cefiderocol MICs [71, 72], which should be determined using broth microdilution methods [73].

Interestingly, a combination of ceftazidime–avibactam and aztreonam may have high in vitro activity against cotrimoxazole- and fluoroquinolone-resistant isolates of *S. maltophilia*, a species that expresses a chromosomal AmpC cephalosporinase susceptible to the inhibitory effect of avibactam in addition to its chromosomal MBL [74, 75].

## In vitro activity against other relevant pathogens responsible for infections in critically ill patients

ESBL are class A serine-β-lactamases that are susceptible to the inhibitory effect of both tazobactam and avibactam. Avibactam also inhibits AmpC cephalosporinases while tazobactam does not. Therefore, both ceftolozane–tazobactam and ceftazidime–avibactam are active against ESBL-producing Enterobacterales without AmpC co-expression but only the latter combination is active against isolates co-producing ESBL and derepressed ampC (e.g., *Enterobacter* spp) [76]. Imipenem–relebactam and meropenem–vaborbactam are highly active against ESBL-producing Enterobacterales due to the intrinsic activity of carbapenems on these pathogens [43, 77].

Gram-positive bacteria and most of cultivable anaerobes are intrinsically resistant to ceftolozane–tazobactam, ceftazidime–avibactam and cefiderocol [78–80]. The activity of imipenem–relebactam and meropenem–vaborbactam on these pathogens does not differ from the one of imipenem and meropenem, respectively.

## Clinical efficacy of cefiderocol and new BL/BLI combinations in DTR-GNB infections

### Data from randomized controlled trials

Most of randomized controlled trials (RCT) evaluating the clinical efficacy and safety of cefiderocol and novel BL/BLI combinations were not focused on DTR-GNB infections and used a carbapenem as comparator [81–86]. Only three RCTs addressed the input of these new agents in the specific context of DTR-GNB infections.

In the TANGO II trial [87], 47 patients with documented CPE infection (mostly KPC-producing *K. pneumoniae*) were treated for 7 to 14 days by either meropenem–vaborbactam or best available therapy (BAT), most often including an aminoglycoside and/or a polymyxin according to susceptibility test results. Rates of clinical success were 66% and 33% (difference, 32%; 95% confidence interval [CI], 3% to 61%) at end of therapy and 59% and 27% (difference, 33%; 95% CI, 5% to 61%) at test-of-cure (ToC) visit for meropenem–vaborbactam and BAT, respectively. Day-28 all-cause mortality rates were 16% and 33% (difference, 18%; 95% CI − 45% to 9%). A composite endpoint of clinical failure and nephrotoxicity occurred less frequently with meropenem–vaborbactam when compared to the BAT arm (31% versus 80%; 95% CI for difference, − 75% to − 23%). In this trial, the efficacy of meropenem–vaborbactam was not evaluated according to the baseline MIC of this combination for the causative pathogen. Only one patient in the BAT arm received ceftazidime–avibactam (single-drug regimen), excluding any comparison between the two BL/BLI combinations.

In the RESTORE-IMI 1 trial [88], 47 patients infected with DTR-GNB (mostly DTR *P. aeruginosa* susceptible to both imipenem–relebactam and colistin) were treated with imipenem–relebactam or an imipenem/colistin combination. The overall rates of clinical success at Day 28 were 71% and 40% in patients receiving imipenem–relebactam and controls, respectively (difference, 26%; 95% CI 1% to 51%) while those of Day-28 all-cause fatality were 10% and 30% (difference, − 17%; 95% CI − 46% to 7%). Drug-related adverse events—especially nephrotoxicity—were considerably more common in the imipenem/colistin arm. Of note, MICs of imipenem–relebactam ranged from 0.5 to 4 mg/L for *P. aeruginosa* isolates; however, whether this baseline MIC impacted the clinical response to the drug was not investigated.

In the CREDIBLE-CR trial [89], 152 patients with a documented DTR-GNB infection (*A. baumannii*, 46%; *K. pneumoniae*, 33%; *P. aeruginosa*, 19%; MIC_90_ of cefiderocol, 1, 4 and 2 mg/L, respectively) received either cefiderocol (single-drug therapy, 85%) or BAT (colistin-based, 67%; combination therapy, 45%) for 5 to 21 days. Overall rates of clinical success at ToC visit were similar in the two arms (53% versus 50%), including in patients with hospital-acquired pneumonia. Rates of microbiological eradication (31% versus 24%) and of relapse (3% versus 11%) were, respectively, higher and lower in the cefiderocol arm. However, the rate of all-cause fatality was numerically higher in the cefiderocol arm at Day 14 (19% versus 12%), Day 28 (25% versus 18%) and at follow-up termination (34% versus 18%). This finding, which could not be linked to any of baseline patient characteristics, was mostly attributable to an excess mortality in patients infected with *A. baumannii* (fatality rate, 49% versus 18% in the cefiderocol and BAT arms, respectively)—no difference was observed between the two arms for patients infected with *P. aeruginosa* or *K. pneumoniae*, except in those with *A. baumannii* co-infection. The baseline MIC value did not correlate with the likelihood of clinical or microbiological failure, which was observed even for isolates with MIC < 0.5 mg/L. Hetero-resistance has been evocated as an underlying mechanism for such observations, notably for *A. baumannii* [90]; nonetheless, whether this phenomenon correlates with the hazard of treatment failure is debated and necessitates further investigations. Following the publication of this trial, the Food and Drug Administration issued a warning statement that advocated for restricting the use of cefiderocol to patients in whom no other therapeutic option is available [91].

### Data from observational and post hoc studies

Regarding CPE, several cohort studies reported clinical success rates above 65–70% with ceftazidime–avibactam for severe infections due to KPC- or OXA-48-like-producing Enterobacterales [92–101] and with meropenem–vaborbactam for severe infections due to KPC-producing Enterobacterales [102–105]. An ancillary study from the CREDIBLE-CR and ASPEK-NP RCT evaluated cefiderocol versus BAT for infections due to MBL-producing Enterobacterales and reported numerically higher rate of clinical success and lower rate of mortality with cefiderocol [106]. Another ancillary study from the same trials and including 10 patients infected with OXA-48-like-producing Enterobacterales reported clinical cure in 7 of them [107]. The clinical efficacy of cefiderocol in CPE infections has also been reported in numerous case-reports and small observational studies [108]. Relevant clinical success rates—similar to those observed with cefiderocol—have equally been observed with the combination of ceftazidime–avibactam and aztreonam for infections due to MBL-producing Enterobacterales [109–111]. No clinical study focused on the efficacy of imipenem–relebactam for infections due to KPC-producing Enterobacterales has been published so far.

To our knowledge, the efficacy of novel BL/BLI combinations in CPE infections has been directly compared in only one study. This work focused on infections due to KPC-producing Enterobacterales (72% of cases) and including roughly half of critically ill patients, ceftazidime–avibactam (*n* = 105) and meropenem–vaborbactam (*n* = 26) showed similar results in terms of clinical and microbiological successes, length of hospital stay, incidence of adverse events, and mortality [112].

Most of cohort studies centered on patients infected with non-MBL-producing DTR *P. aeruginosa* reported clinical success rates above 60% with ceftolozane–tazobactam [92, 113–117] and ceftazidime–avibactam [101, 118–123]. Observational data on the clinical efficacy of imipenem–relebactam are lacking for this patient population.

Lastly, a single-center study with propensity-score analyses using inverse probability of treatment weighting reported a lower mortality rate with cefiderocol versus colistin-based regimen in patients with DTR *A. baumannii* infection (except for those with ventilator-associated pneumonia), contrasting with the results of the CREDIBLE-CR trial [124]. In this work, microbiological failure was twice more frequent in the cefiderocol arm. Nephrotoxicity was more common in the colistin arm [124].

The cohort studies cited above were mostly retrospective and not devoted to critically ill patients. To date, real-life data on the efficacy of these new β-lactams in patients with life-threatening DTR-GNB infection (e.g., septic shock) are dramatically scarce.

## Combination therapy—what clinical evidence?

Enhanced bacterial killing and a reduced risk of resistance emergence are usual arguments for the use of antimicrobial combinations in critically ill patients with GNB infection. Nevertheless, combining antibiotics may also raises concerns related to safety issues including toxicity, drug–drug interactions, and potential ecological impact. Hence, despite decades of extensive research in the field, the benefit-to-risk ratio of combination therapy in this population is still debated, with fragmentary evidence for improved survival only in the most severe presentations [125].

Two meta-analyses of observational studies and RCT found no survival benefit with ceftazidime–avibactam combined with one or more antimicrobials (i.e., fosfomycin, tigecycline, gentamicin, or meropenem) when compared to ceftazidime–avibactam alone for the treatment of DTR-GNB infections [126, 127]. Two large retrospective multicentre cohort studies including patients with infections due to KPC-producing *K. pneumoniae* and published after the above-mentioned meta-analyses yielded similar results [128, 129]; of note, one of them reported a trend toward improved survival with combination therapy in the subgroup of patients with hospital-acquired pneumonia [128]. Clinical evidence related to the potential benefit of combining ceftazidime–avibactam with colistin is limited to case-reports or small case-series, precluding any conclusion to be drawn [95, 130].

A meta-analysis of observational studies demonstrated a significant reduction in all-cause mortality when combining ceftolozane–tazobactam with other antimicrobials in patients with GNB infections—mostly DTR *P. aeruginosa* infections—yet without benefit in terms of clinical cure and microbiological eradication [131]. A subsequent multicentre study focused on DTR *P. aeruginosa* infections in critically ill patients did not confirm this survival benefit [117].

A multi-center retrospective study including 37 patients with severe KPC-producing *K. pneumoniae* infection reported a higher mortality rate with meropenem–vaborbactam combined with another antimicrobial (mostly colistin or fosfomycin) when compared to meropenem–vaborbactam alone; however, patients receiving combination therapy were older, had more comorbidities and presented with higher severity indexes, thereby inducing obvious bias in the interpretation of this result [102].

In a post hoc analysis of the CREDIBLE-CR trial, the proportions of patients with clinical cure and microbiological eradication at ToC visit did not differ between patients receiving cefiderocol as single-drug regimen or in combination; however, only 14 patients received combination therapy [89]. A single-center retrospective study including 16 patients with DTR *A. baumannii* infection showed similar results [132].

To our knowledge, no published data exist to appraise the potential benefit of combining imipenem–relebactam with other antimicrobials for DTR-GNB infections, especially those involving *P. aeruginosa*.

Overall, it remains unclear whether cefiderocol and novel BL/BLI combinations should be associated with antimicrobial agents from other classes to improve patient-centered outcomes in severe DTR-GNB infections, with most of available clinical data coming from retrospective cohort studies. Pending further evidence, combination therapies could be considered in certain situations at high risk for clinical or microbiological failure such as unachievable source control, high bacterial inoculum, or infections due to extensively drug-resistant strains with elevated MICs, as suggested in certain studies evaluating older antimicrobials in DTR-GNB infections [133, 134].

## Clinical pharmacokinetics and optimization of dosing schemes

### New drugs, old PK/PD concepts

**Fig. 1:**
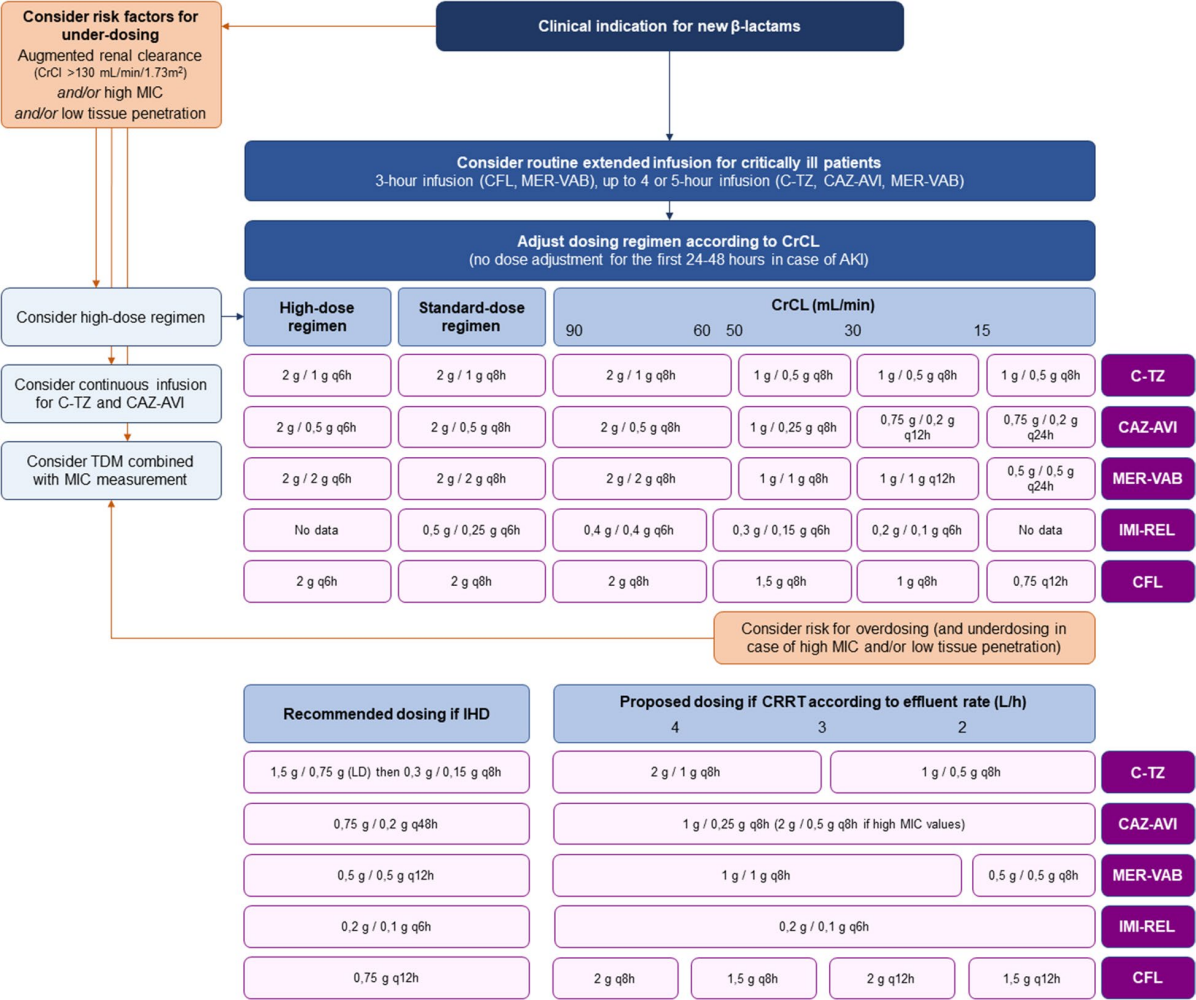
Administration scheme and dosing adjustments of new β-lactam/β-lactamase inhibitor combinations and cefiderocol in critically ill patients. See the text and Table 4 for references. CFL: cefiderocol; CFL: cefiderocol; C-TZ: ceftolozane–tazobactam; CAZ-AVI: ceftazidime–avibactam; MER-VAB: meropenem–vaborbactam; IMI-REL: imipenem–relebactam; CrCl: creatinine clearance; MIC: minimal inhibitory concentration; AKI: acute kidney injury; TDM: therapeutic drug monitoring; IHD: intermittent haemodialysis; CRRT: continuous renal replacement therapy; LD: loading dose

The most efficient pharmacokinetic/pharmacodynamic (PK/PD) index to predict bacterial cell killing with β-lactams is the percentage of the dosing interval during which the concentration of unbound drug exceeds the MIC of the strain (%*f*_T_ > MIC). A reasonable amount of evidence corroborates that *f*_T_ ≥ MIC equal to 100% (i.e., minimal inter-dose concentration [C_min_]/MIC ≥ 1) and even *f*_T_ ≥ 5xMIC equal to 100% (i.e., C_min_/MIC ≥ 5) should be targeted in patients receiving β-lactams for severe infections [135–140], higher C_min_/MIC ratio being linked with enhanced bacterial killing and reduced emergence of resistant mutants [135, 141–143]. Higher blood levels also correlate with improved tissue penetration and bioavailability of the drug at the infection site. As supported by Monte Carlo simulations [144, 145], extending the duration of β-lactam infusion increases drug exposure and allows higher C_min_ targets to be reached, which could translate into improved patient outcome during severe infections [146, 147]. Available data suggest that these concepts apply for new β-lactams and plead for the routine use of 3-h infusion scheme for cefiderocol and meropenem–vaborbactam, and 4- to 6-h infusion scheme for ceftolozane–tazobactam and ceftazidime–avibactam (Fig. 1) [128, 144, 148–151]. The stability of the drug in syringes at room temperature must be considered when using extended or continuous infusion. Recent studies reported a stability of 4 to 8 h for meropenem (in dextrose 5% and normal saline, respectively), 12 h for vaborbactam, and 24 h for ceftazidime–avibactam, ceftolozane–tazobactam, cefiderocol and aztreonam (either in dextrose 5% or normal saline) [152, 153].

### Dosing adjustment in particular situations

New BL/BLI combinations and cefiderocol share similar characteristics with older β-lactams, including high therapeutic indexes, heterogeneous inoculum effect, hydrophilicity, small molecular weights, almost exclusive renal clearance, low protein-binding (except for cefiderocol) implying effective elimination by renal replacement therapy (RRT), and variable tissue diffusion (Table 4) [53, 154]. Dosing adjustment may be required in specific clinical situations to ensure sufficient antibiotic concentration at the infectious site while avoiding toxic levels to be attained [155, 156].

**Table 4:** Available PK/PD data for new β-lactam/β-lactamase inhibitor combinations and cefiderocol

Antibiotic	PK/PD index (preclinical studies)	Molecular weight (Dalton)	Protein binding (%)	Half-life (hours)	V_D_ (liters)	Renal clearance^a^	Elimination rate in RRT^b^	ELF/plasma ratio (%)	CSF/plasma ratio (%)	References
Ceftazidime–avibactam										
Ceftazidime	*f*_T_ > MIC > 50%	637	< 10	HA: 1.5–2 CIP: 4.8	HA: 14–17 CIP: 35	80–90%	55%	HA: 35–52 CIP: 21–43	20–40	[212–219]
Avibactam	*f*T > 1 mg/L > 50%	265	6–8	HA: 1.5–2 CIP: 4.1	HA: 15–25 CIP: 51	> 95%	55%	HA: 35–42 CIP: 22	32	
Ceftolozane–tazobactam										
Ceftolozane	*f*_T_ > MIC > 35–40%	765	16–21	HA: 3.5	HA: 13–18 CIP: 20	> 95%	66%	HA: 48–61 CIP: 50	20	[146, 201, 204–209, 220–225]
Tazobactam	*f*T > 1 mg/L > 20%	322	30	HA: 2.3	HA: 18 CIP: 32	80%	56%	HA: 44–63 CIP: 62	20	
Imipenem–relebactam										
Imipenem	*f*_T_ > MIC > 40%	317	20	HA: 1	HA: 24 CIP: 20	63%	66–87%	HA: 55	8.5	[226–230]
Relebactam	*f*_AUC0-24_/CMI > 8	366	22	HA: 1.2	HA: 19	> 90%	67–87%	HA: 54	NA	
Meropenem–vaborbactam										
Meropenem	*f*_T_ > MIC > 75%	437	2	HA: 1.2	HA: 19 CIP: 16–33	40–60%	38%	HA: 65	8–24	[231–240]
Vaborbactam	*f*_AUC0-24_/MIC > 9	297	33	HA: 1.6	HA: 20.5	75–95%	53%	HA: 79	NA	
Aztreonam–avibactam										
Aztreonam	*f*_T_ > MIC > 50%	435	56	HA: 2.3–2.8	HA: 20	80%	38%	HA: 30	3–52	[218, 219, 241–243]
Avibactam	*f*T > 2–2.5 mg/L > 50%	265	8	HA: 1.5–2	HA: 22	> 95%	55%	HA: 35–42 CIP: 22	32	
Cefiderocol	*f*_T_ > MIC > 75%	752	40–60	HA: 2.7	HA: 18	90%	60%	HA: 22 CIP: 10–55	4–68	[151, 244–253]

PK: pharmacokinetic; PD: pharmacodynamic; V_d_: volume of distribution; RRT: renal replacement therapy; ELF: epithelial lining fluid; CSF: cerebrospinal fluid; MIC: minimal inhibitory concentration; AUC: area under the curve; HA: healthy adult; CIP: critically ill patient; NA: non-available

^a^Unchanged for all; ^b^ intermittent haemodialysis or continuous RRT

First, augmented renal clearance (ARC), consensually defined as a creatinine clearance ≥ 130 mL/min/1.73m^2^, may reduce C_min_ and overall drug exposure, thereby lowering the probability of PK/PD target attainment, especially for bacterial isolates with high MIC values. For instance, through continuous infusion of ceftolozane–tazobactam has been shown to ease PK/PD target attainment in most cases, 4-h extended infusion may be more effective for MIC values ≥ 4 mg/L combined with ARC [144]. Higher-dose regimen appear also needed in this situation (Fig. 1) [149, 151, 157]. Of note, cefiderocol is the only new β-lactam for which high-dose schemes were used in patients with ARC in pivotal randomized studies—further data are needed for new BL/BLI combinations in this population.

Next, while renal dysfunction exposes to β-lactam over-dosing, therapeutic failures have also been reported in this context, raising the question of inadequate PK/PD target attainment [158]. For novel agents as for older β-lactams, recommendations for dose adjustment in case of renal dysfunction are mostly based on data from patients with chronic kidney disease. Because of increased volume of distribution in critically ill patients, possible resolution of acute renal dysfunction within the first following days [155, 159], high therapeutic index and a limited risk of neurotoxicity with short exposure to high doses, a loading dose is recommended regardless of ClCr and dose adjustment should be applied only after the first 24 to 48 h of therapy [150, 154, 160, 161]. Evidence for dosing adjustment of cefiderocol and new BI/BLI combination in critically ill patients receiving RRT is currently scarce; however, data related to older β-lactams suggest that dosing scheme should be adapted to the overall effluent flow rate during continuous RRT while systematic reinjection at the end of the session may ensure the achievement of PK/PD objectives during intermittent hemodialysis [162–165].

Of note, hepatic dysfunction does not induce any clinically relevant variation in β-lactam PK; therefore, no dosing adjustment is required in patients with liver failure.

Data are also lacking to appraise the pharmacokinetic impact of obesity for the new drugs addressed here. Generally, the hydrophilic feature of β-lactams explains the weak variation of their volume of distribution in case of overweight [166, 167]. Whether using adjusted body weight may be beneficial in obese patients remains to be confirmed [166]. Higher dosing could be discussed in cases of morbid obesity and high MIC values [168].

Case-reports and small case-series suggest that extracorporeal membrane oxygenation (ECMO) exerts no major effect on the pharmacokinetics of ceftolozane–tazobactam and cefiderocol, with standard dosing enabling the attainment of usual targets [169–172]. No clinical information is available for other new β-lactams in patients under ECMO.

Through this approach does not appear justified in all critically ill patients [173, 174], therapeutic drug monitoring combined with MIC measurement (to avoid undue dose escalation) could be proposed in those at marked risk for PK/PD impairments (e.g., causative pathogen with high MIC value, ARC, RRT, or low tissue penetration rate). Close collaboration between microbiologist, pharmacist, infectious disease specialist and intensivist is warranted in these situations.

## Safety issues

### Non-ecological adverse events

No apparent over-risk of drug-related adverse events was noticed with cefiderocol or new BL/BLI combinations in RCTs comparing these agents with meropenem, imipenem plus colistin, or BAT [81, 83, 86–89]. Importantly, the hazard of acute kidney injury was higher with BAT in nearly all studies using this regimen as comparator, a finding mostly attributable to colistin-induced nephrotoxicity [87–89, 124]. Encephalopathy may conceivably occur with these drugs as with older β-lactams though it remains to be explored [175, 176]. A recent large-scale pharmacovigilance analysis suggested an over-reporting of agranulocytosis/pancytopenia and acute pancreatitis with ceftolozane–tazobactam and ceftazidime–avibactam, respectively—these observations require confirmation in clinical cohorts [175].

### Impact on the intestinal microbiota

Evidence related to the impact of ceftazidime–avibactam on the gut microbiota is limited to a single study including 12 healthy volunteers receiving standard dosing (2 gr/0.5 gr q8h) for 7 days [177]. This work, based on conventional cultures and not on modern metagenomics approaches, showed a transient decrease in Enterobacterales counts, an increase in the count of *Enterococcus* spp (without return to baseline 14 days after treatment completion in most of volunteers), a sustained drop in the counts of cultivable resident anaerobes and, strikingly, the acquisition of a toxinogenic strain of *Clostridioides difficile* in 5 subjects [177]. This apparent link between ceftazidime–avibactam exposure and *C. difficile* acquisition was not confirmed in clinical studies—indeed, in a multicentre cohort including 203 patients receiving ceftazidime–avibactam, only 3 cases (1.5%) of *C. difficile* infection were documented [123]. To date, no data exist regarding the impact of ceftolozane–tazobactam, imipenem–relebactam, meropenem–vaborbactam or cefiderocol on the gut microbiota. Clinical studies addressing this issue appear extremely complex to set up since almost all patients requiring these drugs present with multiple risk factors for intestinal dysbiosis, including critical illness and prior exposure to other broad-spectrum antimicrobials [178].

### Emergence of resistance under therapy

Treatment-emergent resistance to ceftazidime–avibactam in KPC-producing Enterobacterales mostly results from mutations of the *bla*_KPC-2_ or *bla*_KPC-3_ genes and may be involved in up to 30% of clinical failure or relapse following exposure to this drug [179–184]. Acquired resistance to meropenem–vaborbactam and imipenem–relebactam in CPE appears mostly driven by impermeability ensuing from mutation-induced porin loss—this phenomenon appears rare in patients treated with meropenem–vaborbactam (< 5%) while its incidence has not been precisely described in those receiving imipenem–relebactam [105, 112, 179]. In *P. aeruginosa*, the emergence of resistance to ceftolozane–tazobactam rests on mutation-induced over-expression of the chromosomal *bla*_AmpC_ gene: this mechanism might be involved in up to 50% of patients with microbiological failure at end of therapy, especially in case of intermittent infusion (when compared to extended infusion) and defective source control [185]. However, in a post hoc analysis of the ASPECT-NP RCT including 59 patients receiving ceftolozane–tazobactam for nosocomial pneumonia due to *P. aeruginosa*, only 3 (5%) acquired a ceftolozane–tazobactam-resistant isolate under therapy, all with a new strain (no resistant mutant selection) [186]. Treatment-emergent resistance to imipenem–relebactam in patients infected with CR *P. aeruginosa* has been recently linked with mutations in the MexAB-OprM and/or MexEF-OprN efflux operons [187]—the clinical frequency of this phenomenon is unknown. Lastly, in the CREDIBLE-CR trial, a fourfold or higher increase in baseline cefiderocol MIC values of causative pathogens was observed in 15% of microbiologically evaluable patients receiving this agent—this increase led to values exceeding the EUCAST susceptibility threshold in one third of cases [89].

## Empirical use of cefiderocol and new BL/BLI combinations in critically ill patients

Pending dedicated studies on this issue, several key aspects of antimicrobial stewardship should be taken in account when considering the potential utilization of cefiderocol and new BL/BLI combinations for empirical therapy in patients with suspected DTR-GNB infection. First, the choice of empirical antimicrobials must be a “winning bet” in case of severe infection. Indeed, while delayed appropriate therapy is strongly associated with impaired outcomes in patients with septic shock [188, 189], unnecessary exposure to broad-spectrum antimicrobials may lead to deleterious ecological side-effects (namely, alteration of the host microbiota, acquisition of multidrug-resistant bacteria, and *Clostridioides difficile* infection) and toxic adverse events [190, 191]. Conversely, evidence exists that a restrictive strategy for empirical initiation of broad-spectrum antimicrobials in hemodynamically stable patients with suspected ICU-acquired infection has no negative impact on hospital mortality [192]. Second, the emergence of bacterial resistance under therapy has been described for virtually all antimicrobial agents, including cefiderocol and novel BL/BLI combinations [89, 112, 179, 193, 194]. Hence, a liberal utilization of these new drugs might compromise their activity on CR-GNB. Third, not all ICU patients are at-risk for infection due to DTR-GNB. Identifying such patients is a challenge that can be approached using known risk factors such as recent exposure to carbapenems and other broad-spectrum agents, invasive healthcare procedures, and, most of all, local epidemiology—that is, endemicity or on-going outbreaks, especially in case of defective infection prevention measures. The colonization status is also pivotal since carriage is a prerequisite for subsequent infection; however, through negative sequential surveillance cultures have a high negative predictive value, less than 50% of critically ill individuals colonized with carbapenem-resistant GNB will experience a healthcare-associated infection due to these pathogens during their ICU stay [195–197]. In addition, full antimicrobial susceptibility test results—or, at least, information on the determinants of carbapenem resistance—should be available for clinicians to assist the choice of the most appropriate drug since these agents are not identical with respect to their spectrum of activity and mechanisms of action (Table 1). Multiplex PCR assays enabling species and carbapenemase identification directly from clinical samples in short turn-around time could be useful, but their input warrants further investigation [198]. Of note, these tools are ineffective to detect carbapenem resistance resulting from chromosomal mutations—e.g., in *P. aeruginosa* [199].

Published evidence on the empirical use of new β-lactams in critically ill patients is currently lacking. These agents might be administered empirically in patients at high-risk for DTR-GNB (that is, known carriage or local endemicity with high colonization pressure) and presenting with life-threatening healthcare-associated infection (e.g., septic shock). Every probabilistic prescription should be reevaluated early to avoid unnecessary exposure to these drugs, with prompt de-escalation to a narrower-spectrum regimen whenever possible.

## Should ceftolozane–tazobactam and ceftazidime–avibactam be prescribed as carbapenem-sparing agents in patients infected with ESBL- or AmpC-produding Enterobacterales?

Published RCT have demonstrated the non-inferiority of ceftolozane–tazobactam versus meropenem in terms of clinical cure in patients with complicated intra-abdominal infections (in combination with metronidazole) or nosocomial pneumonia [82, 83]. In this latest trial, ceftolozane–tazobactam was non-inferior to meropenem in patients with pneumonia due to ESBLE or ceftazidime-resistant *P. aeruginosa*, for clinical cure as for Day-28 all-cause mortality [83]. Moreover, a multi-center study including 153 patients with severe ESBLE infections reported an 84%-overall success rate with ceftolozane–tazobactam [200]. Yet, important considerations argue against the use of ceftolozane–tazobactam as a carbapenem-sparing option in patients with ESBLE infections, including the willingness to preserve its efficacy on DTR *P. aeruginosa* [193], the hazard of co-selection of ceftazidime–avibactam resistance in *P. aeruginosa* isolates with treatment-emergent resistance to ceftolozane–tazobactam [201], and the lack of data regarding a potential benefit of ceftolozane–tazobactam versus carbapenems regarding antimicrobial-induced intestinal dysbiosis. Likewise, the results of several RCT [81, 202–207] and a meta-analysis [208] support the non-inferiority of ceftazidime–avibactam versus carbapenems on mortality and/or clinical cure endpoints in complicated urinary tract infections, complicated intra-abdominal infections (in combination with metronidazole) and nosocomial pneumonia, even when focusing on ESBL- or AmpC-producing Enterobacterales [209]. Nevertheless, similar arguments than for ceftolozane–tazobactam argue against the liberal use of ceftazidime–avibactam in these common indications, notably the risk of reduced activity on KPC-producing Enterobacterales resulting from mutant selection [182, 210] or the lack of real-world data demonstrating a more limited impact on commensal microbiotas when compared to carbapenems. As others [1], we believe that the use of these BL/BLI combinations should be restricted to clinical situations in whom no first-line safe options are available—that is, infections due to DTR *P. aeruginosa* plus, for ceftazidime–avibactam only, those due to KPC- or OXA-48-producing Enterobacterales.

## Summary of evidence and research agenda

New BL/BLI combinations and cefiderocol represent long-awaited options for improving the management of DTR-GNB infections. These drugs have demonstrated relevant clinical success rates and a reduced renal risk in most of situations for whom polymyxin- and/or aminoglycoside-based regimen were historically used as last-resort strategies—that is, ceftazidime–avibactam for infections due to KPC- or OXA-48-like-producing Enterobacterales, meropenem–vaborbactam for KPC-producing Enterobacterales, ceftazidime–avibactam/aztreonam combination or cefiderocol for MBL-producing Enterobacterales, and ceftolozane–tazobactam, ceftazidime–avibactam and imipenem–relebactam for non-MBL-producing DTR *P. aeruginosa*. To preserve their efficacy, these drugs should not be used to treat infections due to multidrug-resistant but carbapenem-susceptible GNB (e.g., ESBL-producing Enterobacterales).

Notwithstanding these promising results, limited evidence exists on the use of new β-lactams in critically ill patients with DTR-GNB infection. Several important knowledge gaps warrant urgent investigation in this population, including PK/PD information in particular situations (e.g., pneumonia or other deep-seated infections, RRT and ARC), the benefit of combination therapy for the most severe presentations or DTR-GNB with high MIC values for these new agents, the input of TDM, a precise appraisal of the hazard of treatment-emergent resistance and possible preventive measures, safety analyses (especially for high-dose regimen), the potential usefulness of multiplex PCR assay and other rapid diagnostic tools to rationalize their empirical utilization in ICUs facing endemicity or on-going outbreaks, and optimal treatment durations. Comparative clinical, ecological and medico-economic data are equally needed for situations in whom two or more of these agents exhibit in vitro activity against the causative pathogen. Further studies addressing the aforementioned issues will help better defining the positioning and appropriate administration scheme of these new β-lactams in critically ill patients.

## Supplementary Information

**Additional file 1.** Criteria for literature search.

## Data Availability

Not applicable.

## Abbreviations

ARC: Augmented renal clearance
BAT: Best available therapy
BL/BLI: β-Lactam/β-lactamase inhibitor combination
CI: Confidence interval
C_min_: Minimal inter-dose concentration
CPE: Carbapenemase-producing Enterobacterales
DTR: Difficult-to-treat resistance
eCDC: European Centre for Disease Control and Prevention
ESBL: Extended-spectrum β-lactamase
EUCAST: European Committee on Antimicrobial Susceptibility Testing
GES: *Guyana* Extended-spectrum β-lactamase
GNB: Gram-negative bacteria
ICU: Intensive care unit
IMP: Imipenemase
KPC: *Klebsiella pneumoniae* Carbapenemase
MBL: Metallo-β-lactamase
MIC: Minimal inhibitory concentration
NDM: New-Delhi metallo-β-lactamase
OXA: Oxacillinase
PCR: Polymerase chain reaction
PD: Pharmacodynamic
PK: Pharmacokinetic
RCT: Randomized controlled trial
RRT: Renal replacement therapy
TDM: Therapeutic drug monitoring
ToC: Test-of-cure
VIM: Verona integron-encoded metallo-β-lactamase
