# Supplementary material for: Rationale and evidence for the use of new beta-lactam/beta-lactamase inhibitor combinations and cefiderocol in critically ill patients
Source: Ann Intensive Care. 2023 Jul 18;13:65. doi: 10.1186/s13613-023-01153-6 (PMC10354316; doi:10.1186/s13613-023-01153-6)
Supplement: Supplementary file 1 — Additional file 1. Criteria for literature search. [file 13613_2023_1153_MOESM1_ESM.docx]

**Rational and evidence for the use of new beta-lactam/beta-lactamase inhibitor combinations and cefiderocol in critically ill patients**

François Barbier, MD PhD; Sami Hraiech, MD PhD; Solen Kernéis, MD PhD; Nathanaël Veluppillai, MD; Olivier Pajot, MD; Julien Poissy, MD PhD; Damien Roux, MD PhD; Jean-Ralph Zahar, MD PhD, on behalf of the French Intensive Care Society

**Key-words used for literature search**

We systematically searched PubMed and the Cochrane Library database from inception to November 15, 2022 using the following key-words:

Combined with

Gram-negative bacteria

OR

Enterobacterales

OR

*Klebsiella pneumonia*

OR

*Escherichia coli*

OR

*Enterobacter*

OR

*Pseudomonas aeruginosa*

OR

*Acinetobacter baumannii*

OR

*Stenotrophomonas maltophilia*

OR

Carbapenem-resistant

OR

Difficult-to-treat resistance

OR

Carbapenemase

OR

Metallo-β-lactamase

OR

Infection

OR

Critically ill

OR

Intensive care unit

OR

Outcome

OR

Pharmacokinetics

OR

Combination therapy

OR

Adverse event

OR

Toxicity

OR

Gut microbiota

Ceftolozane-tazobactam

OR

Ceftazidime-avibactam

OR

Imipenem-relebactam

OR

Meropenem-vaborbactam

OR

Cefiderocol

We manually searched the reference lists of the included studies and systematic reviews to select additional relevant articles. Studies published in languages other than English were not retained.
